# Eye tracking: the silver bullet of competency assessment in medical image interpretation?

**DOI:** 10.1007/s40037-019-0506-5

**Published:** 2019-04-04

**Authors:** Ellen M Kok

**Affiliations:** Department of Education, Faculty of Social Sciences, Utrecht University, Utrecht, The Netherlands

‘Eye tracking may be valuable for informing assessments of competency progression during medical education and training’ [1]. Brunye et al. [1] and other researchers (e.g. [2]) have made this suggestion to argue the relevance of using eye tracking to investigate medical image interpretation. Eye tracking is a technique to measure the movements of the eyes to investigate what a person looks at, for how long, and in what order [3]. It can help us to go beyond mere outcome measures (i.e. the percentage of cases correctly diagnosed) and provide an insight into the process of medical image interpretation. Whereas previous research has shown that eye tracking is a very useful tool to investigate the interpretation of medical images (such as angiograms), the field is not yet at a point where eye tracking can be used for competency assessment in clinical practice. In this commentary on ‘Eye-tracking during dynamic medical image interpretation: a pilot feasibility study comparing novice vs expert cardiologists’ [1] I discuss what eye tracking could add to competency assessment, which eye-tracking measures are potential markers of expertise, and what is still needed before they can be used for competency assessment.

What is the added value of eye tracking? Can we not just ask people what they look at? As it turns out, we cannot. People have a limited ability to report on their own viewing behaviour [4]. Radiologists reading digital breast tomosynthesis images, for example, reported that they restricted their eye movements to a region of breast tissue while scrolling through the depth of the image [5]. In reality, they moved their eyes over the whole image while scrolling through depth, which is potentially a less effective strategy [6]. Eye tracking can thus provide objective information about viewing behaviour that cannot be verbally reported and could, as such, contribute to competency assessment.

Which measures qualify as useful markers of expertise? Eye-tracking data is often parsed into fixations (when the eye is relatively still and takes in information) and saccades (jumps between fixations). Measures are, for example, the duration, velocity, and number of fixations and saccades [7]. Not all of those measures are equally useful as markers of expertise. For example, Brunye and colleagues [1] have found that experts have, on average, a lower number of fixations than novices. This measure does not exploit the possibilities of eye tracking: It just reflects the fact that experts perform the task more quickly, which can also be observed with a simple (and cheap) stopwatch. So what measures do exploit the possibilities of eye tracking? It is often found that, with increasing expertise, average fixation duration decreases, the length of saccades increases, the number and duration of fixations on relevant versus irrelevant information increases, and the time to first fixation of relevant information decreases [8–10]. Yet, some studies do not find expertise differences in these measures and some find the opposite pattern of results. Then how could we use and interpret these measures as markers of expertise?

Only measures that are grounded in theory can be meaningfully interpreted. For example, the time to first fixation of relevant information and the average length of saccades are commonly used as measures of holistic processing: Experts are thought to quickly form a holistic representation of the image, which guides their subsequent viewing behaviour. This allows them to quickly look at relevant information, whereas novices use a search-to-find approach. These two measures can thus reflect how well a resident can already form a holistic impression.

At the same time, the above-mentioned measures are not suitable for all stimuli. For example, in earlier research, we did not find that experts made longer saccades towards abnormalities than novices when they inspected chest radiographs showing global diseases (i.e. the disease affects most of the lungs) [11]. In this situation, there is no ‘relevant’ information to quickly jump to, since most of the image is relevant for diagnosis. Likewise, forming a holistic impression of dynamic stimuli such as angiograms is probably different from forming a holistic impression of chest radiographs. Finding universal markers of expertise is thus impossible: Measures should always be chosen and interpreted in the context of a theoretical framework and the specific stimulus. This also means that researchers should not restrict themselves to the above-mentioned measures. Other measures can be much better suited to a theoretical concept or stimulus.

What else is needed for competency assessment? Ideally, eye-movement measures should not just have different average values between different expertise levels, but their values should also predict performance if they were to be used for competency assessment. Unfortunately, a correlation between eye-tracking measures and performance is not always found. For example, in earlier research, we found that experts showed more systematic viewing behaviour than novices, but we found no significant correlation between how systematic novices looked and their performance [12]. For competency assessment, studies are thus needed that establish which measures predict performance.

Furthermore, for measures that do predict performance, such as the average time to first fixation in a mammography study [13], it is often only known that they differ between experts and novices, but their detailed development over time is still unknown. Thus, for competency assessment, longitudinal eye-tracking studies are required to delineate how, for example, the average time to first fixation of an abnormality changes with increasing expertise.

In conclusion, eye tracking could have added value for competency assessment. A large amount of literature has shown eye-tracking measures that could be markers of expertise, but universal markers of expertise are not feasible. Measures should always be chosen and interpreted in the context of a theoretical framework and specific stimulus. Furthermore, before eye tracking can be implemented for competency assessment, we need studies that detail which measures predict performance and longitudinal studies that establish in detail how they develop over time.
